# *Rhamnusalaternus* Plant: Extraction of Bioactive Fractions and Evaluation of Their Pharmacological and Phytochemical Properties

**DOI:** 10.3390/antiox10020300

**Published:** 2021-02-16

**Authors:** Amine Nekkaa, Akila Benaissa, Fabrice Mutelet, Laetitia Canabady-Rochelle

**Affiliations:** 1 Process Engineering Laboratory for Sustainable Development and Health Products, Department of Process Engineering, National Polytechnic School of Constantine—Malek Bennabi, Constantine 25000, Algeria; 2 Laboratoire Réactions et Génie des Procédés, CNRS, Université de Lorraine, F-54000 Nancy, France; fabrice.mutelet@univ-lorraine.fr; 3 Laboratory of Process Engineering for the Environment (LIPE), Department of Pharmaceutical Engineering, Faculty of Process Engineering, Salah Boubnider University, Constantine 3, Constantine 25000, Algeria; akila.benaissa@univ-constantine3.dz

**Keywords:** *Rhamnus alaternus*, extraction processes, phytochemistry, ethnopharmacology, phytotherapy, toxicity, bioactive compounds

## Abstract

*Rhamnus alaternus*, is a wild-growing shrub, belonging to the *Rhamnaceae* family. Widely distributed in the Mediterranean basin*, R. alaternus* is used in the usual medicine in numerous countries, mostly Tunisia, Algeria, Morocco, Spain, France, Italy, and Croatia. A large number of disorders—including dermatological complications, diabetes, hepatitis, and goiter problems—can be treated by the various parts of *R. alaternus* (i.e., roots, bark, berries, and leaves). Several bioactive compounds were isolated from *R. alaternus*, including flavonoids, anthocyanins, and anthraquinones, and showed several effects such as antioxidant, antihyperlipidemic, antigenotoxic, antimutagenic, antimicrobial, and antiproliferative. This review summarizes the updated information concerning the botanical description, distribution, extraction processes applied on *R. alaternus*, and its ethnopharmacology, toxicity, phytochemistry, and pharmacological effects.

## 1. Introduction

*Rhamnus* species are considered as medicinal plants. Indeed, these sources of natural compounds possess pharmacological activities, and are used for their curative effects to treat some symptoms and diseases [1,2,3,4,5,6,7]. During the past few years, several studies highlighted the potential efficacy of *Rhamnus* species in many areas [8,9,10,11]. Among these naturally available species, the *Rhamnus alaternus* plant (*R. alaternus*) is commonly recognized as a 5-meter-tall shrub, and is distributed throughout the Mediterranean area [12,13,14,15] including North Algeria, Tunisia, and Morocco [16,17]. This plant widely grows in a Mediterranean climate with hot and dry summer and winter period is moderate to cold [14,18,19].

The *Rhamnus alaternus* plant, the so-called "Imlilesse or Safir" in the North of Algeria, has been traditionally used for a long time in various medicine areas as infusion notably for its gastric, hypotensive, purgative, laxative, diuretic, antihypertensive, hepatoprotective, and digestive effects and finally to treat dermatological complications [20]. Such biological activities would be related to the natural presence of beneficial compounds as evidenced by many experimental studies, that pointed out that *R. alaternus* contains important metabolites—such as flavonoids, coumarins, glycosides, tannins, anthraquinones, and polyphenolic compounds [21,22]. Some of these molecules were isolated from *Rhamnus alaternus* using various extraction processes (i.e., maceration, decoction, hydrodistillation, soxhlet, ultrasonic extraction) and demonstrated various pharmacological properties including antihyperlipidemic, antioxidant, antigenotoxic, antiproliferative, and antimutagenic activities. Some biological activities, especially antibacterial and antiproliferative effects were reviewed elsewhere [8,20,22,23,24].

To date, to the author’s knowledge, there are no accurate published reviews concerning *Rhamnus alaternus*. In this current review, we compiled and described various extraction methods of bioactive compounds applied on various parts of this plant. Besides therapeutic effects, phytochemical, and pharmacological activities of *R. alaternus* are presented. The exploitation and investigation of potential beneficial effects of *Rhamnus alaternus* are summarized here in order to guide the research strategy for its industrial application.

## 2. Botanical Data

### 2.1. Geographical Distribution

Common in wild, *Rhamnus alaternus* growths generally between evergreen shrubs of the Mediterranean region, especially in a climate with discontinuous rains during winter. With such characteristics, *R. alaternus* is a very important species of the Mediterranean basin, where it is well acclimated to high solar radiation [25,26]. *Rhamnus alaternus* is widely distributed and grows naturally in a large part of the littoral and islands of the Mediterranean. In France, this plant is mainly found in the South departments such as Isère, Ardèche, Aveyron, Maine-et-Loire, in Vienne but also in Brittany [27]. In addition, this plant growths in Corsica, Algeria, and Northern Tunisia [22,28,29,30].

### 2.2. Botanical Description 

**Figure 1:**
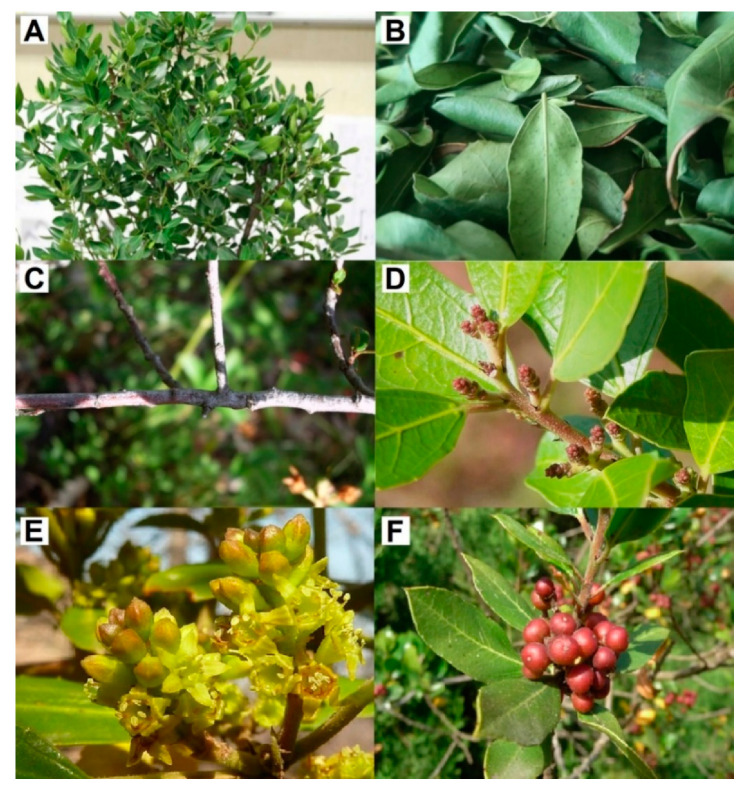
(**A**) *Rhamnus alaternus* plant with a focus on its different aerial parts: (**B**) leaves, (**C**) stem, (**D**) pods, (**E**) flowers, and (**F**) berries.

In North Africa, *Rhamnus alaternus* has several names such as Am’lile’ce, M’lila, Soitfaïr, Oud El-khir, or Safir, and is commonly known as Meliles in Berber language [31,32,33]. *R. alaternus* is also called Buckthorn in English, Nerprun in French, Kreülzdorn in German, Aladierna, Cosco Unia, or Sanguino de Andalucia in Spanish, and Alaterno or Legno Puzzo in Italian [34]. Concerning its botanical classification, *R. alaternus* belongs to the *Magnoliophyta* division, the *Magnoliopsida* class, the *Rhamnales* order, the *Rhamnaceae family,* the *Reynosia* Genus and the *Rhamnus alaternus* species [35]. In addition, this plant has various synonyms including *R. a*. var *angustifolia* DC, *R. a*. var *b*alearica** DC, *R. a*. var *hispanica* DC, and *R. a*. var *vulgaris* DC [36]. *R. alaternus* is a small shrub of about 5-meter-tall (Figure 1A–D). Its flowers are fecundated by insects or with the help of wind [37,38] and get yellow-green from January until the end of April (Figure 1E), with a top in mid-February [39]. Then puffy and black fruits are produced, and mature between late spring and early summer, each one containing between 2 and 5 red berry seeds with on average 2.5 mm width, 4.6 mm length, and 9.1 mg weight as maximum (Figure 1F) [38,40]. Seeds are surrounded within a pericarp, which opens up once dried [38], and represents an important trophic source for birds and small mammals [41]. Usually germinated in 3 to 4 weeks between 7.5 and 24 °C, the seeds remain viable for several years in storage [40].

## 3. Extraction Processes Investigated in *R. alaternus*

### 3.1. Main Processes Applied

The bioactive compounds present in *Rhamnus alaternus* plant can be extracted by using various techniques described in Figure S1 (see Supplementary Materials) with the critical parameters of each extraction process. Besides, all techniques used for the extraction of bioactive compounds from different parts of *R. alaternus* were reported with the solvents used for isolating the targeted compounds, and their respective pharmacological and biological activities including: antioxidant, antimicrobial, antigenotoxic, and antiproliferative activities (Table 1) [42,43,44,45,46]. Also, the chemical structure of the molecules isolated from this plant are presented in Figure S2 (see Supplementary Materials).

### 3.2. Maceration and Decoction

Among all the reported extraction processes, maceration and decoction are the most commonly applied methods to extract biomolecules [59,60]. The maceration method is vastly used in research domain of medicinal plants. These methods are described and explained by authors in numerous references [60,61,62]. The decoction method uses the same concept as maceration. Yet, for the decoction, the plant powder is boiled in a specific volume of water for a defined time, then cooled and filtered, while in maceration, the plant powder is mixed with extraction solvent at room temperature [61,62].

In 2015, Boussahel and colleagues investigated the flavonoid profile obtained by decoction and maceration of aqueous and methanolic extracts, obtained from *R. alaternus* bark and determined their antioxidant activity. Former authors observed that the methanolic maceration was better for extracting flavonoids than both aqueous maceration and aqueous decoction [21]. In another study, Berroukche and coworkers compared the same former extraction processes applied on leaves of *Rhamnus alaternus* to study their antioxidant and hepatoprotective effects [49]. The results of their investigation proved that leaves extracts obtained by maceration were better and more effective than those obtained by decoction in terms of bioactivity. Also, Moussi and colleagues investigated the antioxidant activity of the methanolic extract from *R. alaternus* leaves and reported that leaves extract from this plant can be used for formulating pharmaceutical products for various diseases [48]. The composition of *R. alaternus* roots, bark and leaves extracts was extensively investigated using the maceration method [18,20,22,24,47,50,51,52,55,57,58]. The same extraction techniques were used by Tacherfiout and colleagues, who investigated the antihyperlipidemic effect of leaves extracts of *Rhamnus alaternus* [23].

### 3.3. Soxhlet Extraction 

Soxhlet extraction is one of the reference methods used for extraction of bioactive compounds [45,46]. This technique is known for its simplicity, a low cost with a minimal solvent consumption compared to other conventional methods. In this technique, the ground sample is placed in a ‘thimble’ made from cellulose, itself located in a thimble chamber of the Soxhlet system, in the presence of extraction solvents. These latter ones are heated in a distillation flask, and evaporated inside the sample thimble. After the liquid reaches the siphon arm, the liquid content empties back into the distillation flask and the operation is continued until extraction is complete [61,63]. 

Numerous studies focused on the Soxhlet extraction of some bioactive molecules from different parts of *R. alaternus*. To date, various solvents were used in this extraction method such as ethanol, ethyl acetate, methanol, chloroform, etc. [64]. By using Soxhlet extraction and fractionation, Ben Ammar et al., succeeded in isolating three bioactive substances from leaves extracts of this plant endowed with high antioxidant and free radical scavenging capacities [50]. In another study, Bhouri and colleagues investigated the antioxidant and antigenotoxic activities of three bioactive compounds extracted from the leaves of *R. alaternus* using soxhlet extraction method [24]. The results of their investigation proved that compounds obtained from *R. alaternus* leaves are phytopharmaceutical molecules of interest. Other studies revealed that different extracts of *Rhamnus alaternus* obtained by soxhlet has antigenotoxic, antimutagenic, and antimicrobial activities [20,51,52,53,56,58].

### 3.4. Ultrasonic Assisted Extraction

Ultrasonic assisted extraction (UAE) has the main advantage to extract bioactive compounds from plants without impacting on their functional properties [65]. This technique depends on the use of ultrasounds power ranging from 20 kHz to 2000 kHz [61,66]. UAE is an important extraction method applied in food processing. It uses ultrasonic waves to create cavitation bubbles in order to increase the mass transport between solvent and plant cells, and leads to fast and effective extraction of compounds from plants [46,61,67].

In 2013, Kosalec and co-workers used ultrasonic extraction technique and proved that *R. alaternus* was an important source of anthraquinones and other bioactive substances [8]. In addition to three other *Rhamnus* species investigated, former authors reported the anthraquinone profile of the *R. alaternus* bark extracts, characterized with antioxidant and antimicrobial activities. 

### 3.5. Hydrodistillation

Hydrodistillation (HD) is a frequently used method for extraction of volatile compounds. In a reactor, the plant material is immerged in boiling water mostly at atmospheric pressure; the volatile compounds are moved away with the water steam up to the condenser and collected after decantation [68,69]. This technique depends mainly on the distillation time, which impacts the solvent penetration into the plant matrix, and favors the diffusion of substances into the solvent of extraction [70,71].

In their study, Berka and colleagues attempted to isolate essential oil from aerial parts of *R. alaternus* using hydrodistillation extraction [54]. This work was the only one to focus on the essential oil of *Rhamnus alaternus*, which is constituted of a complex mixture of 94 constituents. 

## 4. Phytochemistry

### 4.1. Generalities

The phytochemical investigations of *Rhamnus alaternus* extracts led to the isolation of various classes of natural bioactive compounds, and evidenced the richness in secondary metabolites of this medicinal plant. These bioactive compounds included flavonoids, tannins, anthraquinones, anthocyanins, anthocyanidins, and other compounds [48,72], isolated from various parts of *R. alaternus* such as barks, leaves, roots, and berries extracts. The investigation of these compounds was limited to qualitative studies. The most important classes of phytochemicals identified in *R. alaternus* are summarized (Table 2) with their main chemical compounds, whose structures are presented in Supplementary Data. Each class of compounds is reviewed hereafter.

### 4.2. Flavonoids

Flavonoids, whose skeleton is based on about 15-carbon and is composed of two benzene rings [73,74], gather the bioactive compounds with low molecular weight (286–610 g/mol).

These flavonoids are the most active constituents of *Rhamnus alaternus*. Among these isolated compounds, literature reports quercitin-3-0-rhamninoside, kaempferol-3-0-rhamninoside, quercitin-4’-0-rhamninoside, kaempferol-4’-0-rhamninoside, rhamnetin-3-0-rhamninoside, rhamnocitrin-3-0-rhamninoside, and rhamnocitrin-4’-0-rhamninoside (Figure S2(1)–(7)), identified from green fruits of *Rhamnus alaternus* [55]. Other flavonols —including quercitin, kaempferol, isorhamnetin, rhamnetin, rhamnazin (Figure S2(8)–(12))—were extracted by maceration, from methanolic and aqueous extracts from Algerian *Rhamnus alaternus* barks [21,55]. More, flavonols such as kaempferol 3-0-β-isorhamninoside, rhamnocitrine 3-0-β-isorhamninoside, and rhamnetin-3-0-isorhamninoside (Figure S2(13)–(15)) were also isolated from *Rhamnus alaternus’s* leaves by Soxhlet extraction method [24,50]. Furthermore, other valuable bioactive compounds were isolated from *R. alaternus’* leaves such as kaempferol 3-O acetyl-rhamnoside and quercetin-3-rhamnoside [23,48].

### 4.3. Anthraquinone Compounds

Anthraquinones are aromatic organic compounds with the 9,10-anthracenedione core [75]. These ones, including three new Anthraquinones, were isolated from the extracts of various parts collected from *Rhamnus alaternus* (i.e., leaves, barks, and roots). Among anthraquinones, rhein, chrysophanol, and physcion (Figure S2(17)–(19)) were also isolated from the bark extract of *R. alaternus* by ultrasonic extraction [8]. Furthermore, 1,4,6,8 tetrahydroxy-3 methyl anthraquinone 1-O-β-D-glucopyranosyl-4,6-di-O-α-L-rhamnopyranoside, 1,2,6,8 tetrahydroxy-3 methyl anthraquinone 8-O-β-D-glucopyranoside and 1, 6 dihydroxy-3 methyl 6 [2′-Me (heptoxy)] anthraquinone (Figure S2(20)–(22)) were identified from various parts of *Rhamnus alaternus* such as leaves, bark and roots [16].

### 4.4. Anthocyanin Constituents

Anthocyanins are structurally related to anthocyanidins (parent class of flavonoids) and are also derived from the 2-phenylbenzopyrilium ion [76,77].

The extracts of *Rhamnus alaternus*’ berries showed many compounds of high nutritional values and were rich in diverse anthocyanins and anthocyanidins constituents, such as delphinidin 3-rutinoside, delphinidin 3-glucoside, delphinidin, cyanidin 3-rutiniside, cyanidin 3-glucoside, cyanidin, pelargonidin 3-rutinoside, pelargonidin 3-glucoside, pelargonidin, petunidin, peonidin, and malvidin [18] (Figure S2(27)–(44)). 

## 5. Biological Properties

### 5.1. Ethnopharmacology 

*Rhamnus alaternus* has extensively been used in medicine in Algeria and many other North African countries [22,58]. This plant was empirically used as a laxative, purgative, diuretic, antihypertensive, and depurative [22,50]. In North African countries, the decoction of the aerial parts of *R. alaternus* such as bark is used against certain dermatological and hepatic diseases [22]. This plant is also used for the treatment of diabetes [57]. More recently, Zeouk and colleagues nicely reviewed the traditional uses and pharmacological aspect of *R. alaternus* [78].

### 5.2. Pharmacological Activities

#### 5.2.1. Antihyperlipidemic Activity

Lipids, among them cholesterol, play a fundamental role in the structure of membranes and in many biological activities in the human heart’s health. Yet, an increase in the concentration of lipids—and particularly of plasma cholesterol beyond the dose required daily—presents a major risk of progression of heart and vascular diseases, including coronary heart diseases and strokes [79,80]. Hyperlipidemia is characterized by decreased levels of high-density lipoprotein cholesterol (HDL-cholesterol) and elevated levels of total cholesterol (TC), phospholipids, triglyceride (TG), low density lipoprotein cholesterol (LDL-cholesterol) and very low-density lipoprotein cholesterol (VLDL-cholesterol) into the bloodstream [81,82,83]. According to a World Health Organization report, the cardiovascular diseases caused by hyperlipidemia—including high blood cholesterol—are responsible of about 4.4 million deaths per year [84]. 

The antihyperlipidemic activity of *Rhamnus alaternus* was first investigated by Tacherfiout et al. [23]. According to these authors, the leaves’ extract of *R. alaternus* contains flavonoids that possess the ability to reduce the intracellular lipids concentration and to increase the oxidation of fatty acids in HepG2 cells. Similarly, the flavonoids and flavonoids derivatives from *R. alaternus* leaves showed a similar positive impact on murine preadipocyte 3T3-L1 cellular model.

#### 5.2.2. Antioxidant Activity

Natural antioxidants are bioactive substances extracted from plants that reduce damages due to reactive oxygen species, in both food and human body. Antioxidants might reduce the cancer risks and protect against different diseases. The antioxidant capacity is a generic term that gathers various mechanisms such as radical-scavenging activity, that can be evaluated by DPPH (2,2 -diphenyl-1- picrylhydrazyle) or ABTS assay (2,2’-azinobis (3-ethylbenzthiazoline-6-sulfonic acid)), and other antioxidant tests carried out in vitro [85,86,87,88,89] notably involving inhibition of lipid peroxidation or metal-chelation. 

Natural compounds, including polyphenols, have various pharmacological activities and biological effects, such as antioxidant properties [90,91,92,93,94,95], and are known for their protective effects against many oxidative stress-related diseases [96,97,98,99,100].

The antioxidant properties of *R. alaternus* are associated with the presence of numerous constituents, such as flavonoids and anthraquinones [48,50]. Numerous studies focused on the antioxidant activity of natural substances extracted from *R. alaternus* [16,31,50]. Most of these studies evaluated the antioxidant properties using in vitro methods such as the 1,1-diphenyl-2picrylhydrazyl (DPPH) radical [98]. Zeouk and colleagues [47] demonstrated that ethanolic extracts of *R. alaternus* obtained by maceration from leaves harvested in the Atlas Mountains of Morocco possess an interesting antioxidant capacity. Indeed, using DPPH antioxidant test, former authors isolated three fractions with a very good antioxidant activity (IC_50_ = 58.00 ± 7.00 µg/mL), compared with Butyl hydroxytoluene BHT (positive control; IC_50_ = 31.00 ± 3.00 µg/mL).

In 2015, Boussahel and co-workers investigated the antioxidant activity of the aqueous and methanolic extracts of *R. alaternus* barks, obtained either by maceration or by decoction [21]. They evaluated the antioxidant properties while coupling various biochemical tests: radical scavenging tests, such as the DPPH and the ABTS tests (2,2’-azinobis-(3-ethylbenzothiazine-6-sulfonic acid), the ferric reducing ability test that uses the FRAP reagent (ferric reducing antioxidant power) and the determination of oxygen radical absorbance capacity (ORAC) assay [21]. Former authors focused first on the solubility of flavonoids in organic and aqueous solvents, and evidenced that the flavonoids of *Rhamnus alaternus* bark were more easily dissolved in methanol than in water. Concerning their antioxidant effect, the methanolic extract presented a higher content of flavonol (51.17 ± 0.41 µg QE/mg) compared to the aqueous one (24.10 ± 0.85 µg QE/mg) using quercetin as reference substance. Hence, the methanolic extract was more bioactive whatever the chemical assays used, in particular for the test of flavonoid quantification described by Tamiano et al. [101]. Also, the same extract showed the highest Trolox equivalents antioxidant capacity (TEAC = 0.75 ± 0.001 mmol TE/g) using Trolox as reference substance [21].

In another study [24], the antioxidant properties of two flavonoids isolated from *Rhamnus alaternus* leaves using Soxhlet extraction method were evaluated using the ABTS assay. The two isolated constituents—the kaempferol 3-O-β-isorhamninoside and rhamnocitrin 3-O-β-isorhamninoside (Figure S2(13),(14))—showed an interesting inhibitory activity against superoxide anion at a dose of 150 µg/assay. Former authors determined that these two compounds exhibited a potential radical scavenging activity with IC_50_ values, ranging from 18.75 to 22.5 µg/mL. In comparison, the Trolox reference exhibited an IC_50_ value of 0.2 µg/mL. Furthermore, Moussi et al. [48] reported the antioxidant properties of various fractions obtained from *R. alaternus* leaves by maceration and found an interesting radical scavenging capacity for all fractions separated from the methanolic extract. Besides, the antioxidant activity evaluated by DPPH assay presented a high percentage of DPPH radical inhibition (90.36 ± 0.45%). 

More recently, Ben Ammar et al. [16] isolated three new bioactive substances from various parts of *Rhamnus alaternus* (i.e., leaves, roots, and barks). They evaluated the DPPH radical scavenging activity of these new identified anthraquinones: alaternoside A, alaternoside B, and alaternoside C. These anthraquinones showed a high free radical scavenging capacity, specially alaternoside C, with an IC_50_ value of 9.46 µg/mL and presented a higher effect than the positive reference (i.e., Vitamin E). Table 3 summarizes the antioxidant activity of *Rhamnus alaternus* collected from different geographical regions, according to the part of the plant investigated and the biological test set up.

According to the data concerning the extraction process of bioactive compounds from different parts of *R. alaternus* and the antioxidant activity of the corresponding fractions, the maceration of the leaves gave an extract, rich in bioactive substances, with the highest antioxidant activity. 

Besides, the inhibition of lipid peroxidation is another indicator of antioxidant activity. Indeed, the lipid peroxidation affects unsaturated lipids, and is related to the presence of radical species [98]. In the long-term, lipid peroxidation process could lead to the development of various diseases such as diabetes and several cancers [99,102]. The anti-lipid peroxidation activity of various extracts from *R. alaternus*, produced using the Soxhlet extraction method, was estimated by calculating the values of malondialdehyde (MDA) in cultured K562 human chronic myelogenous leukemia cells [52]. *Rhamnus alaternus* extracts containing total oligomer flavonoids (TOF) and ethyl acetate (EA) inhibited lipid peroxidation at a concentration comprised within 200–800 µg/mL, the best activity being observed at the highest concentration (800 µg/mL). In this study, the IC_50_ values of TOF and EA extracts were determined at 196 and 265 µg/mL, respectively. In comparison, a value of 17 µg/mL was obtained for vitamin C, used as reference substance.

**Table 3:** Antioxidant activity of *Rhamnus alaternus* collected from various countries.

Parts of Plant	Analytical Method	Values	Country	Reference
Leaves	DPPH assay	1.5–38 µg/mL equivalent vitamin C (fractions)	Tunisia	[50]
		12.60–90.81%; BHT is the positive control (fraction)	Algeria	[48]
		8.22 ± 0.01µg/mL; BHT is the positive control (extract)	Algeria	[42]
		07.76–38.87% (fractions)	Morocco	[47]
	TEAC assay	18.75–22.5 µg /mL equivalent of Trolox (fractions)	Tunisia	[103]
		18.75 µg/mL 22.5 µg/mL equivalent of Trolox (fraction)	Tunisia	[24]
	FRAP assay	300–368 µg/mL equivalent of Trolox (fractions)	Tunisia	[31]
Mixture of Leaves, Bark and Roots	DPPH assay	2.35–58 µg/mL Vitamin E is the positive control (fraction)	Tunisia	[16]
Leaves Root	DPPH assay	18.84 µg/mL 7.21 µg/mL, α-tocopherol is the positive control (extract)	Tunisia	[22]
Bark	DPPH assay ORAC assay FRAP assay TEAC assay	0.39–0.61 mmol TE/g 3.96–6.55 mmol TE/g 1.24–1.72 mmol Fe^2+/^g 0.65–0.75 mmol TE/g (extract)	Algeria	[21]
	DPPH assay β-carotene-linoleic acid assay Reducing power assay SRP Chelating activity	78.7 ± 3.16 µg/mL 250 ± 6.84 µg/mL Ascorbic acid, quercetin and BHT are used as positive controls (extract) 0.91 ± 0.01 mg^−1^ 1760 ± 60.7 µg/mL	Croatia	[8]
Arial part	DPPH assay	52.32–87.34% α-tocopherol is the positive control (extract)	Tunisia	[51]

TEAC, Trolox Equivalent Antioxidant Capacity assay; DPPH, 1,1-diphenyl-2-picrylhydrazyl radical; TE, Trolox Equivalent; ORAC, Oxygen Radical Absorption Capacity; BHT, Butyl hydroxytoluene; FRAP, Ferric reducing antioxidant power.

#### 5.2.3. Antiproliferative Activity

Every year in developed countries, many people die because of cancer [104,105]. Many effective anticancer drugs contain biomolecules such as flavonoids, an important class of natural substances found in numerous plants, among them *R. alaternus*. These molecules are characterized by their antiproliferative activities, which are related to their properties to prevent or delay the growth and spread of cells, especially malignant cells, into surrounding tissues. Hence, these molecules could be either used as traditional drugs, or pills once encapsulated [106,107,108,109,110].

The antiproliferative test determines the effect of an investigated biomolecule or extract to prevent the growth of a given cell type. The objective of this assay is to determine the inhibitory concentration (IC_50_) of the investigated biomolecule, i.e., the concentration that inhibits half’s proliferation of the overall cell defined [111,112,113,114].

The antiproliferative effect of roots and leaves extracts obtained from *R. alaternus* maceration was investigated against K562 human cell line and L1210 mouse lymphoma cells, at various concentrations comprised between 100 and 800 µg/mL [22]. The proliferation of these leukemia cells was followed by the MTT assay, which uses the tetrazolium salt (3-(4,5-dimethylthiazol-2-yl)-2,5-diphenyltetrazolium bromide) (MTT) as reagent [115]. The leaves and roots extracts from *R. alaternus* showed interesting antiproliferative, and dose-dependent effects. The root extract was more effective than the leaves one, on both types of leukemia cells. Indeed, concerning the K562 human cell, the IC_50_ values of roots and leaves extracts were determined at 165 and 260.69 µg/mL, respectively. Concerning the L1210 cells, the IC_50_ values of roots and leaves extracts were determined at 210.73 and 343.10 µg/mL, respectively, in the presence of α-tocopherol as positive control. According to these former data, the K562 cell line showed a higher sensitivity to the inhibitory effect of the tested extracts.

#### 5.2.4. Antimicrobial Activity

The antimicrobial activity concerns all active substances or agents that kill or inhibit the growth of bacteria [116]. A large number of plants have always been used to treat prevalent infectious diseases in humans [117,118,119,120], these ones being caused by various micro-organisms, including bacteria. Hence, plants endowed with antimicrobial activity are often used as a part of the usual treatment of various diseases [121,122,123].

Many methods are reported in the literature for the evaluation or detection of antimicrobial effect. The disc diffusion method is a qualitative method since it only gives a trend about the presence of antimicrobial activity of the investigated substances [124,125,126]. The principle of this method is based on the surface culturing of inoculated microbes, which are exposed to small disks containing a quantity of extracts. If the extract has the capacity to inhibit the bacterial growth, it results in a zone of inhibition around the disk after incubation. On the contrary, the dilution method is a quantitative test: it can determine the level of microbial resistance to an extract or an antimicrobial agent by making serial dilutions of the tested agent, in order to define the minimal inhibitory concentration (MIC).

Ben Ammar and colleagues (2007) investigated the antimicrobial activity of aerial parts of various *R. alaternus* extracts obtained by Soxhlet extraction. Such extracts were tested against two Gram-positive bacteria, both from the American type culture collection (ATCC): *Staphylococcus aureus* and *Enterococcus faecalis*. In addition, their antimicrobial activities were tested on three Gram-negative bacteria as well: *Escherichia coli* ATCC, *Salmonella enteritidis* ATCC and *Salmonella typhimurium*. The antimicrobial activity of *R. alaternus* was determined using the micro-dilution method, the range of concentration varying between 50 µg/mL and 6 mg/mL. According to the investigated bacterial species, the MIC values varied from 62.5 µg/mL up to 6 mg/mL, while the minimal bactericidal concentration (MBC) values varied from 1.75 μg/mL to more than 6 mg/mL. The authors observed an important antimicrobial activity for the ethyl acetate (EA) and the total oligomer flavonoids (TOF) extracts, in addition to the most concentrated fraction in flavonoids obtained, the so-called A_2_ fraction [20].

In 2013, Kosalec and colleagues [8] investigated the antimicrobial activity of *R. alaternus* in addition to three other *Rhamnus* species. The methanolic bark extracts obtained by ultrasonic assisted extraction was tested against various bacterial strains: *Escherichia coli* ATCC 10535, *Staphylococcus aureus* ATCC 6538, *Microsporum gypseum* MFBF 3, *Pseudomonas aeruginosa* ATCC 27853, *Candida albicans* ATCC 10231, and *Aspergillus niger* ATCC 16404. The MIC values were determined using the micro-dilution method, with ethanol 96% as negative control; all extracts from *Rhamnus species*, notably the *R. alaternus* one, exhibited an interesting antimicrobial activity against the previous microorganisms.

More recently, Zeouk et al. (2019) investigated the antimicrobial activity of *Rhamnus alaternus* [127] against various staphylococcal strains. After the ethanolic maceration, leaves’ extracts were investigated in a range of concentration varying between 0.5 and 16 mg/mL; with other antibiotics in the strain’s antibiogram, the ampicillin (100 µg/mL) and distillated water were respectively used as positive and negative control. With various resistance intensity, this former extract showed anti-staphylococcal activity notably against *Staphylococcus epidermidis* strains, *Staphylococcus aureus*, and its clinical isolate. Whatever the strain investigated, the MIC values varied between 0.5 and 2 mg/mL. 

From Table 1, we can conclude that the arial parts extracts (i.e., leaves, bark, and berries), obtained by Soxhlet extraction, showed an interesting number of bioactive compounds with the highest antimicrobial activity against various bacterial strains. 

#### 5.2.5. Toxicity

Cytotoxicity is defined as the property of a chemical or a biological agent to be toxic towards cells [128]. Hence, cytotoxicity tests are very important in the biomedical field [129]. The cytotoxicity of various extracts and fractions from *Rhamnus alaternus* plant was investigated on the human chronic myelogenous K562 cells and the murine lymphocytic L1210 leukemia cells, using the 3(4,5-dimethylthiazol-2-yl)2,5-diphenyl-tetrazolium bromide (MTT) colorimetric assay [22]. In the presence of *R. alaternus* leaves and roots extract, former authors observed variations in the measured absorption of blue formazan produced from the MTT reduction by the mitochondrial dehydrogenase. Thus, these extracts have not a cytotoxic response. On the contrary, the cytotoxicity of leaves extract was lower than the one of roots extract on K562 and L1210 cells line.

In 2015, Boussahel and colleagues evaluated the cytotoxicity of bark extracts of *Rhamnus alaternus* on the human leukemia U937 cells and peripheral blood mononuclear cells (PBMCs), using the trypan blue assay. The bark methanolic extract was very toxic for U937 cells compared to the bark aqueous extract obtained by maceration and the infusion bark extract, the IC_50_ values measured being of 6.39, 76.74, and 84.65 µg/mL, respectively. In comparison, the value of Taxol, a reference cytotoxic compound, was 2.47 µg/mL. Besides, the extracts of *R. alaternus* were not toxic for normal PBMC cells line, when the methanolic, aqueous and traditional extracts had an IC_50_ value of 220.35, 38.81, and 29.35 µg/mL, respectively [21].

In 2015, a Tunisian man diagnosed with renal failure and rhabdomyolysis was intoxicated by the daily drink of *Rhamnus alaternus* roots solution for six months, which was used as a traditional treatment of diabetes [57]. Hence, Elyebderi and colleagues (2017) classified *Rhamnus alaternus* as a toxic plant at certain conditions of use in folk medicine, such as its daily consumption of leaves infusion [130]. 

However, the cytotoxic effect of crude methanolic extract (CME) of *Rhamnus alaternus’* leaves was investigated on hepatic cells (HepG2) by the lactate dehydrogenase (LDH) release assay [23]. In this latter study, the CME, used up to 500 µg/mL, did not exhibit any toxicity effect.

#### 5.2.6. Antigenotoxic Activity

The genotoxicity describes the damages of the genetic information inside cells, caused by chemical agents, able to induce cancer. The genotoxicity assays, either carried out in vitro and/or in vivo, aim to discover biomolecules, which can reduce damages on cells [131,132]. Thus, the antigenotoxic activity represents the potential of a bioactive compound to reduce or inhibit the DNA damage generated by various genotoxic agents such as alkylating and exogenous agents [133].

The genotoxicity was measured by the Son of Sevenless (SOS) chromotest microplate assay using the *E. coli* strain [134,135,136]. This quantitative bacterial colorimetric assay enables to determine the antigenotoxicity effect of the plant extracts against the toxic effect induced by the aflatoxin B1 [134,137,138]. 

In 2008, Ben Ammar and colleagues investigated the antigenotoxic activity of *R. alaternus’* leaves extracts obtained by Soxhlet extraction on *Escherichia coli* PQ37 strain [53]. First, they injected the mutagens dose of aflatoxin B1 (AFB1) and nifuroxazide at 10 and 20 µg/assay, respectively. The aflatoxin was used as positive control at 10 µg/assay while the negative control did not contain neither AFB1 nor extracts. No toxicity was observed on *Escherichia coli* PQ37 strain since these concentrations were just below the ultimate genotoxic effect. Besides, the nifuroxazide induction factor (IF) decreased down to 46.6% in the presence of *R. alaternus* extracts with SOS chromotest [53]. Thus, *R. alaternus* leaves extracts using Soxhlet technique contain bioactive compounds with antigenotoxic properties, which contribute to the inhibitory effect of mutagens.

In another study, Ben Ammar and colleagues observed that *R. alaternus* arial part extracts reduced the genotoxicity induced by AFB1 and nifuroxazide mutagens used at 10 and 20 µg/assay, respectively. The high genotoxic-reducing percentage was comprised between 79% and 90% for the three extract concentrations investigated (10, 50, and 250 µg/assay). The AFB1 mutagen was used as positive control, while the negative control did not contain neither extracts nor mutagen AFB1 [58]. In 2011, Bhouri and coworkers evaluated the antigenotoxic activity of two flavonoids isolated from *R. alaternus* leaves using Soxhlet extraction (i.e., kaempferol 3-O-β-isorhamninoside and rhamnocitrin 3-O-β-isorhamninoside), on *E. coli* PQ37 [24]. Former authors led the SOS chromotest with two positive controls—i.e., nifuroxazide and aflatoxin B1—used at 10 µg/assay and 5 µg/assay, respectively. The assay carried out in absence of both aflatoxin B1 and extracts constituted the negative control. For the three flavonoid concentrations studied (1, 5, and 10 µg/assay), the antigenotoxic activity of rhamnocitrin 3-O-β-isorhamninoside (Figure S2(14)) was higher than the one determined for kaempferol 3-O-β-isorhamninoside (Figure S2(13)). 

According to the studies, which focused on the antigenotoxic activity of various parts from *R. alaternus*, a good antigenotoxic activity was present in leaves extracts from *Rhamnus alaternus* using Soxhlet extraction.

#### 5.2.7. Antimutagenic Activity 

The antimutagen agents reduce the genotoxic activity of mutagens such as intercalating and deaminating agents, which can increase the rate of mutation into cells [139,140,141]. The antimutagenic activity of roots and leaves extracts from *Rhamnus alaternus* was determined by the biological Ames assay in order to determine the mutagenic potential of its compounds with sodium azide mutagen [142,143]. 

The antimutagenicity test of roots and leaves’ extracts obtained from *R. alaternus* maceration was carried out in the presence of two strains of *Salmonella Typhimurium* (i.e., TA1535 and TA100) and with a sodium azide concentration of 1.5 µg/plate as mutagen inductor. Sodium azide and spontaneous revertants were used as controls [22]. These *R. alaternus* extracts indicated a result of 434 ± 5 and 51 ± 3 revertants/plate for roots, and 362 ± 6 and 442 ± 7 revertants/plate for leaves extracts with TA1535 and TA100 strains, respectively. The incubation of various doses of *R. alaternus* extracts (i.e., roots, leaves) with *S. typhimurium* TA100 strain evidenced that the leaves’ extract was more efficient to reduce the sodium azide-induced mutagenicity than the root’s one. The incubation of the *Rhamnus alaternus* roots and leaves extracts at the concentration of 5 µg/plate was also investigated in the presence of the *Salmonella Typhimurium* TA1535 strain. For this latter bacterial strain, the roots extract reduced more significantly the sodium azide-induced mutagenicity than the leave’s extract used at the same concentration. 

In another study [51], the authors investigated the antimutagenic activity of leaves extracts by the Ames assay, using the mutagen Aflatoxin B1 (AFB1) at a concentration of 10 µg/plate. This former experiment was carried out with two strains of *Salmonella Typhimurium* (i.e., TA98 and TA100) in the presence of various extracts, and spontaneous revertant was used as control. Petroleum ether, chloroform, methanol, aqueous, and total oligomers flavonoids (TOF) extracts obtained by *R. alaternus* maceration were investigated at various doses (10, 50, and 250 µg/plate) and remarkably reduced the AFB1-induced mutagenicity. In this study, the ethyl acetate fraction obtained from the *R. alaternus* aqueous extract was the most effective at a dose of 250 µg/plate. At such dose, the inhibition percentage of mutagenicity was determined by the Ames assay up to 78% for the TA98 strain. 

The studies carried out by Ben Ammar and colleagues [22,51] showed that leaves, bark, and roots extracts obtained by maceration or Soxhlet extraction present a good antimutagenic activity.

## 6. Conclusions

For the first time, to the author’s knowledge, the various extraction processes applied to *Rhamnus alaternus* in order to obtain bioactive compounds were reviewed here, and related to their biological activities. In this present work, we discuss relevant information concerning this plant, its pharmacological effects, phytochemical profiles, and cytotoxicity of different parts of *R. alaternus* when extracted using different processes (i.e., maceration, decoction, Soxhlet, ultrasonic assisted extraction, and hydrodistillation). Furthermore, the antioxidant, antigenotoxic, and antimicrobial activities of *R. alaternus* were reported. Besides, the natural substances isolated and identified from this plant were presented. *R. alaternus* contains many phytochemical compounds (i.e., flavonoids, tannins, and anthocyanins), which are endowed with important medicinal potentials. These aforementioned compounds render the *Rhamnus alaternus* a suitable source to be used for its natural therapeutic substances. In addition, a large number of pharmacological studies are still insufficient to determine the effects and beneficial therapeutic properties of *R. alaternus*. Yet, this plant may help in the discovery of new bioactive substances for treatment of various digestive diseases and health problems.

## Figures and Tables

**Table 1:** Bioactive compounds extracted from different parts of *R. alaternus* according to the process of extraction involved and their pharmacological activities.

Part of Plant	Extraction Method		Solvent(s) Used	Bioactive Compounds (or Groups)	Pharmacological Activities	Reference
	Maceration		Methanol	Kaempferol hexoside, rhamnocitrin, kaempferol 3-O acetyl-rhamnoside, quercetin, pilosin hexoside, pilosin, apigenin glucoside, rhamnocitrin hexoside, rhamnetin hexoside, kaempferol	Antihyperlipidemic acitivity	[23]
			Ethanol, distillated water, methanol, ethyl acetate	Emodin, kaempferol	Antioxidant activity	[47]
			Methanol	Rutin, antraquinones, quercetin-3-rhamnoside, kaempferol, gallic acid, p-coumaric acid, ferulic acid, luteolin	Antioxidant activity	[48]
	Maceration/ Decoction		Distilled water	NA	Hepatoprotective effects	[49]
	Soxhlet extraction/ Maceration		Methanol, petroleum ether, chloroform, ethyl acetate water/acetone	Rhamnetin-3-O-isorhamninoside, kaempferol 3-O-isorhamninoside, rhamnocitrin- 3-O-isorhamninoside	Antioxidant activity	[50]
Leaves			Methanol, chloroform, petroleum ether, ethyl acetate water, acetone	Kaempferol 3-O-β-isorhamninoside, rhamnocitrin 3-O-β-isorhamninoside	Antioxidant activity Antigenotoxic activity	[24]
			Petroleum ether, chloroform, ethyl acetate, methanol, butanol water, acetone	Coumarins, flavonoids, anthraquinones, tannins	Antimicrobial activity	[20]
			Petroleum ether, chloroform, ethyl acetate, methanol, water, acetone	Flavonoids, anthraquinones, tannins, sterols, coumarins	Antigenotoxic activity Antimutagenic activity	[51]
			Ethyl acetate, water, acetone, chloroform	Flavonoids	NA	[52]
	Soxhlet extraction		Chloroform, water, petroleum ether, ethyl acetate, dimethyl sulfoxide, butanol	Flavonoids, phenols	Antigenotoxic activity Antimutagenic activity	[53]
	Hydrodistillation extraction		Water	Oxygenated monoterpenes hydrocarbons, oxygenated diterpenes hydrocarbons, oxygenated Sesquiterpenes hydrocarbons, sesquiterpenes hydrocarbons, monoterpenes hydrocarbons, aliphatic hydrocarbons, fatty acids	NA	[54]
Berries	Maceration	Methanol		Malvidin, delphinidin 3-rutinoside, cyandin, petunidin 3-glucoside, petunidin, delphinidin 3-glucoside, pelagonidin, malvidin 3-rutinoside, peonidin 3-rutinoside, peonidin, cyanidin 3-rutinoside, delphindin peonidin 3-glucoside, malvidin 3-glucoside, cyanidin 3-glucoside, petunidin 3-rutinoside, pelargonidin 3-rutinoside,	NA	[18]
		Methanol, water		Quercetin, rhamnazin-3-O rhamninoside, rhamnazin, quercetin-4′-O-rhamninoside, rhamnetin, kaempferol-4′-O-rhamninoside, isorhamnetin, rhamnocitrin, kaempferol, rhamnocitrin-3-O-rhamninoside, quercetin-3-O-rhamninoside, rhamnetin-3-O-rhamninoside, rhamnocitrin-4′-O-rhamninoside, kaempferol-3-O-rhamninoside	NA	[55]
	Soxhlet extraction	Petroleum ether, methanol		Emodin	NA	[56]
Bark	Maceration/Decoction	Methanol, water		Quercetin, kaempferol, rhamnocitrin, rhamnetin,	Antioxidant activity	[21]
	Ultrasonic extraction	Methanol, ethyl acetate		Flavonoids, aloe-emodin, rhein, emodin, chrysophanol, physcion	Antioxidant activity Antimicrobial activity	[8]
Roots	Decoction	Chloroform, dichloromethane, ethyl acetate		Rhein, physcion, aloe-emodin	NA	[57]
Leaves root bark	Maceration	Methanol, butanol		Flavonoids, coumarins, anthraquinones, sterols	Antiproliferative activity Antimutagenic activity	[22]
	NA	NA		Emodin- 6-O-α-L-rhamnoside, β-sitosterol, physcion-8-O-rutinoside, kaempferol-7-methylether. 1, 6 dihydroxy-3 methyl 6 [2′-Me (heptoxy)] anthraquinone. β-sitosterol-3-O-glycoside. 1,4,6,8 tetrahydroxy-3 methyl anthraquinone 1-O-β-D-glucopyranosyl-4,6-di-O-α-L rhamnopyranoside. 1,2,6,8 tetrahydroxy-3 methyl anthraquinone 8-O-β-D-glucopyranoside	Antioxidant activity	[16]
Aerial part	Soxhlet extraction/Maceration	Methanol, ethyl acetate, chloroform water, acetone		Flavonoids, tannins	Antigenotoxic activity	[58]

NA, not available.

**Table 2:** Compounds isolated from *Rhamnus alaternus.*

Compound Class	Compound	Compound Number *	Reference
Flavonoids	Quercetin-3-O-rhamninoside	**1**	[48,55]
	Kaempferol-3-O-rhamninoside	**2**	[55]
	Quercetin-4′-O-rhamninoside	**3**	[55]
	Kaempferol-4′-O-rhamninoside	**4**	[55]
	Rhamnetin-3-O-rhamninoside	**5**	[55]
	Rhamnocitrin-3-O-rhamninoside	**6**	[55]
	Rhamnocitrin-4′-O-rhamninoside	**7**	[55]
	Kaempferol	**8**	[21,23,47,48,55]
	Quercetin	**9**	[21,23,55]
	Isorhamnetin	**10**	[21,55]
	Rhamnetin	**11**	[21,55]
	Rhamnazin	**12**	[55]
	Kaempferol-3-O-isorhamninoside	**13**	[23,24,50]
	Rhamnocitrin-3-O-isorhamninoside	**14**	[24,50]
	Rhamnetin-3-O-isorhamninoside	**15**	[50]
Anthraquinones	Emodin	**16**	[8,47,56]
	Rhein	**17**	[8,57]
	Chrysophanol	**18**	[8]
	Physcion	**19**	[8,57]
	1,4,6,8 tetrahydroxy-3 methyl anthraquinone	**20**	[16]
	1-O-β-D-glucopyranosyl-4,6-di-O-α-L-rhamnopyranoside		
	1,2,6,8 tetrahydroxy-3 methyl anthraquinone 8-O-β-D-glucopyranoside	**21**	[16]
	1, 6 dihydroxy-3 methyl 6 [2′-Me (heptoxy)] anthraquinone		
	Physcion-3-O-β-rutinoside	**22**	[16]
	Emodin-6O-α-L-rhamnopyranoside	**23**	[16]
	β-sitosterol	**24**	[16]
	β-sitosterol-3-O-β-D-glycopyranoside	**25**	[16]
		**26**	[16]
Anthocyanins	Cyanidin 3-rutinoside	**27**	[18]
	Petunidin 3-rutinoside	**28**	[18]
	Delphinidin 3-rutinoside	**29**	[18]
	Pelargonidin 3-rutinoside	**30**	[18]
	Peonidin 3-rutinoside	**31**	[18]
	Malvidin 3-rutinoside	**32**	[18]
	Delphinidin 3-glucoside	**33**	[18]
	Cyanidin 3-glucoside	**34**	[18]
	Petunidin 3-glucoside	**35**	[18]
	Pelargonidin 3-glucoside	**36**	[18]
	Peonidin 3-glucoside	**37**	[18]
	Malvidin 3-glucoside	**38**	[18]
	Delphindin	**39**	[18]
	Cyandin	**40**	[18]
	Petunidin	**41**	[18]
	Pelagonidin	**42**	[18]
	Peonidin	**43**	[18]
	Malvidin	**44**	[18]

* These numbers refer to the chemical structures plotted in Supplementary Data (Figure S2).

## Supplementary Materials

The following are available online at https://www.mdpi.com/2076-3921/10/2/300/s1, Figure S1: Extraction processes commonly applied on *R. alaternus*. Figure S2: Biomolecules found in *Rhamnus alaternus.* Table S1: name of compounds of Figure S2.
