# Supplementary material for: Rhamnusalaternus Plant: Extraction of Bioactive Fractions and Evaluation of Their Pharmacological and Phytochemical Properties
Source: Antioxidants (Basel). 2021 Feb 16;10(2):300. doi: 10.3390/antiox10020300 (PMC7920288; doi:10.3390/antiox10020300)
Supplement: Supplementary file 1 [file antioxidants-10-00300-s001.pdf]

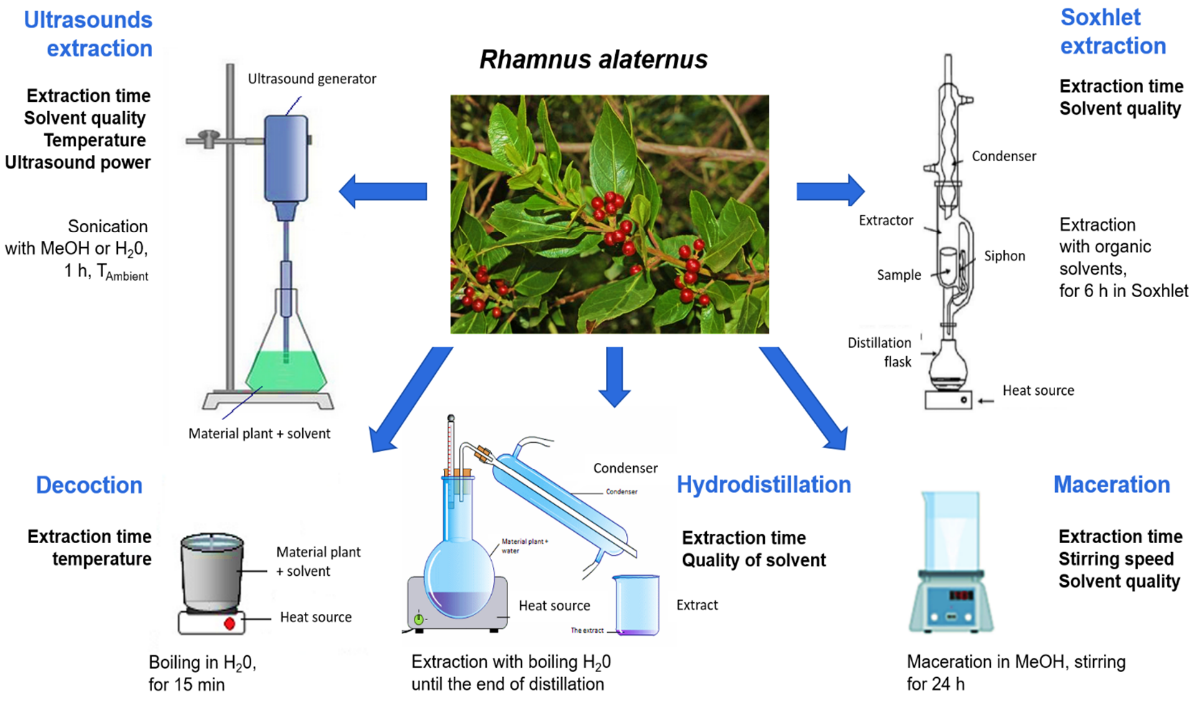

Figure S1. Extraction processes commonly applied on *R. alaternus*.

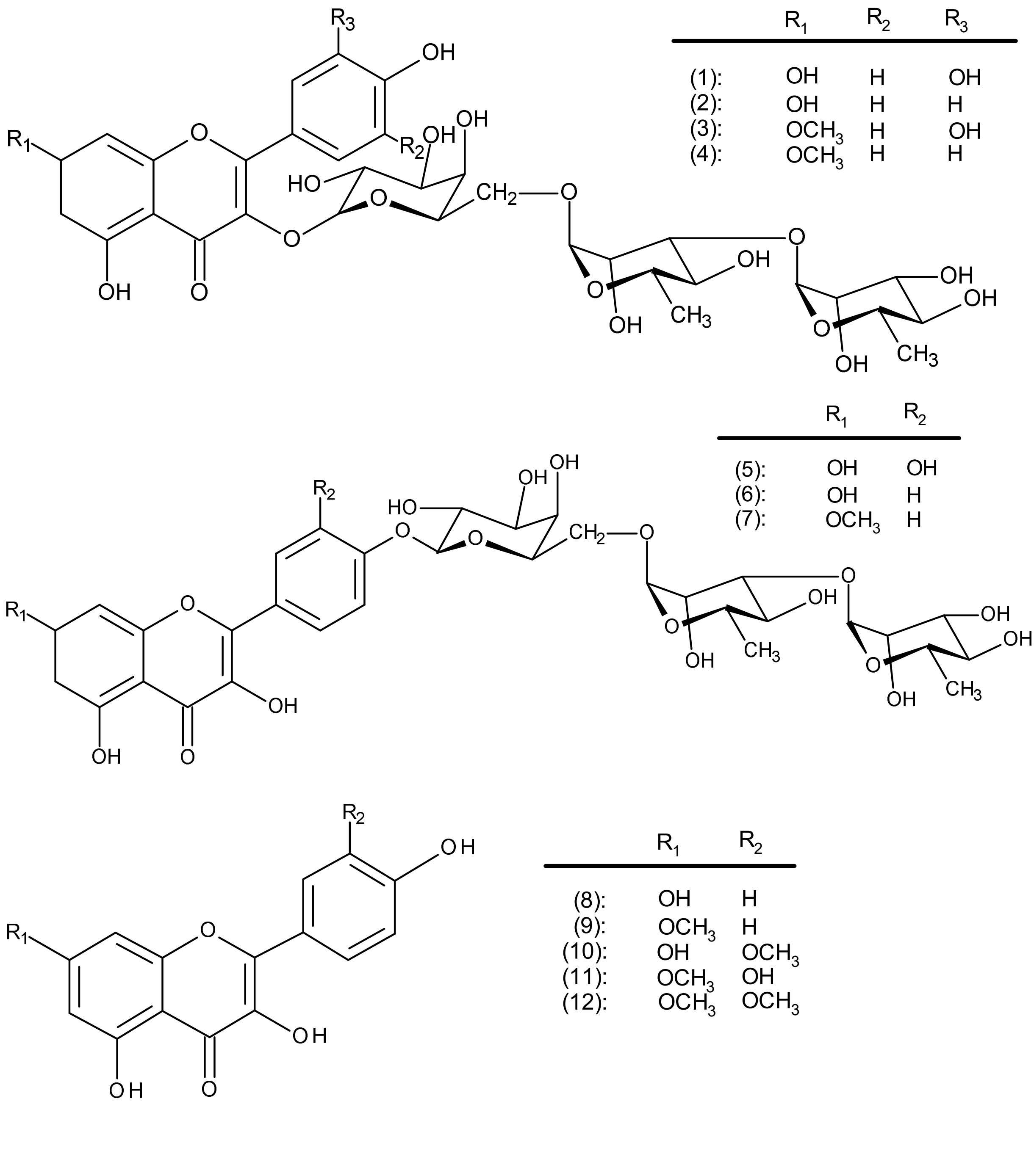

	R1	R2	R3
(1):	OH	H	OH
(2):	OH	H	H
(3):	OCH_3	H	OH
(4):	OCH_3	H	H

	R1	R2
(5):	OH	OH
(6):	OH	H
(7):	OCH_3	H

	R1	R2
(8):	OH	H
(9):	OCH_3	H
(10):	OH	OCH_3
(11):	OCH_3	OH
(12):	OCH_3	OCH_3

Figure S2. Biomolecules found in *Rhamnus alaternus*.

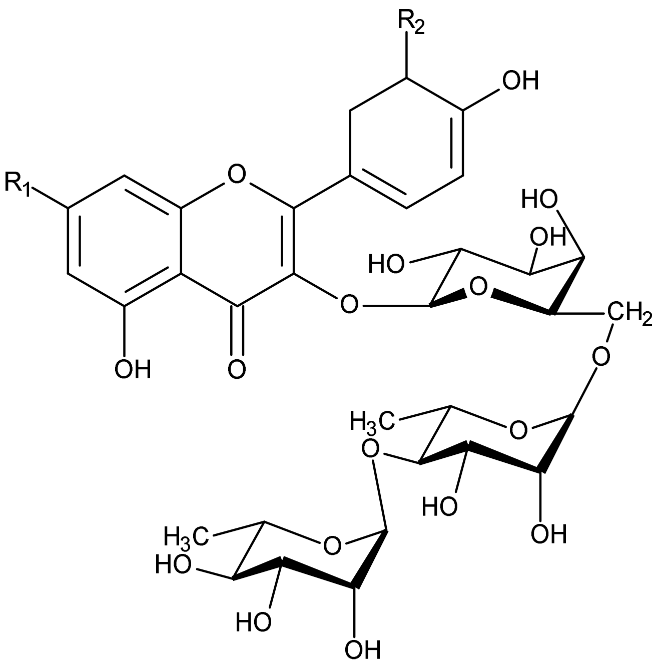

	R_1	R_2
(13):	OH	H
(14):	OCH_3	H
(15):	OCH_3	OH

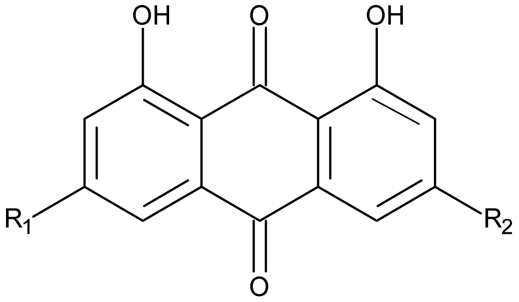

	R_1	R_2
(16):	CH_3	OH
(17):	H	COOH
(18):	H	CH_3
(19):	CH_3	OCH_3

Figure S2. *(Continued)*

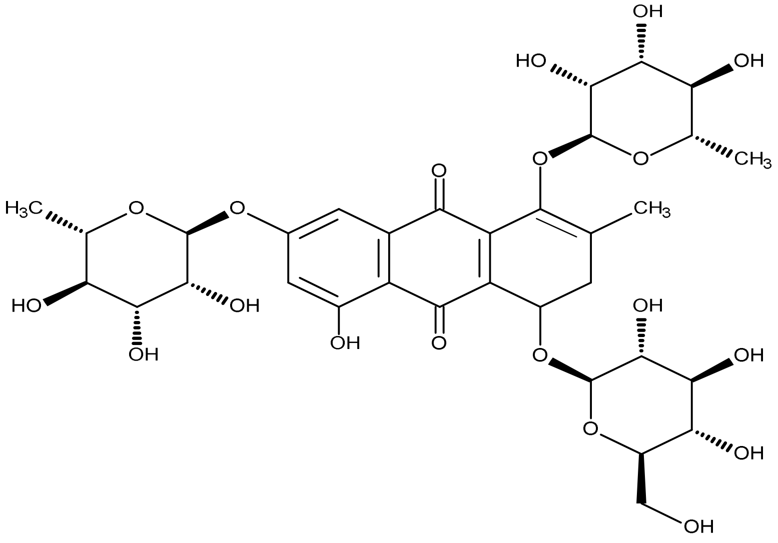

(20)

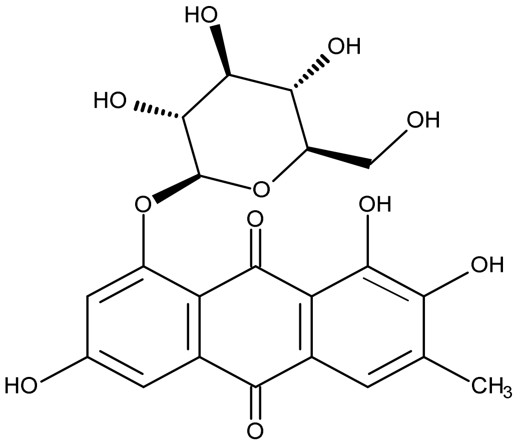

(21)

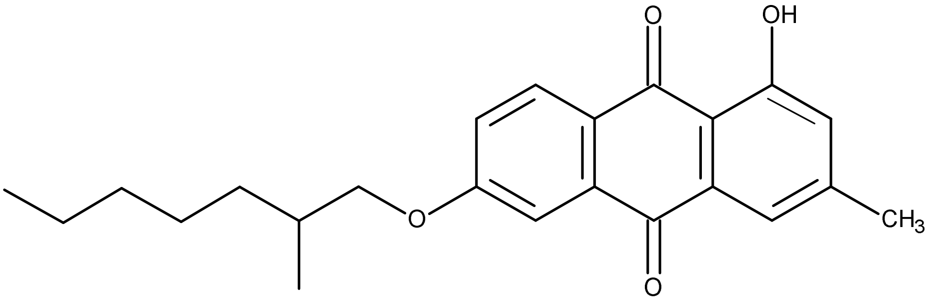

(22)

Figure S2. *(Continued)*

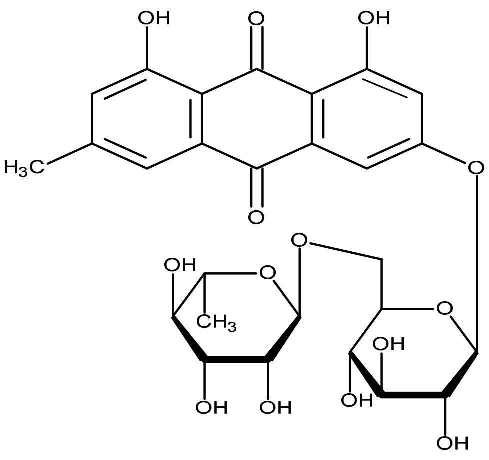

(23)

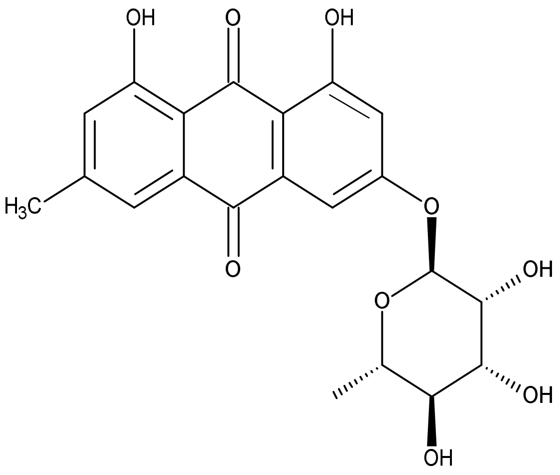

(24)

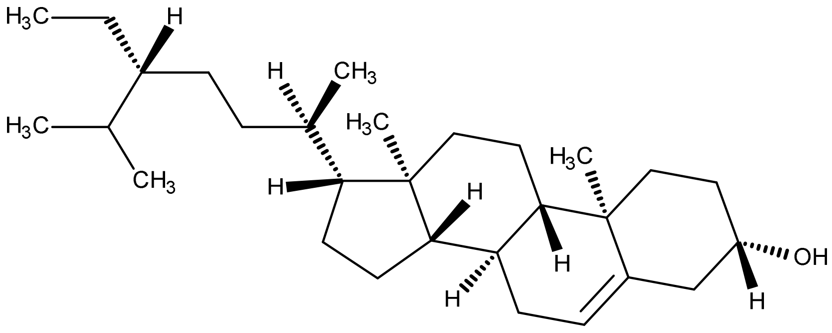

(25)

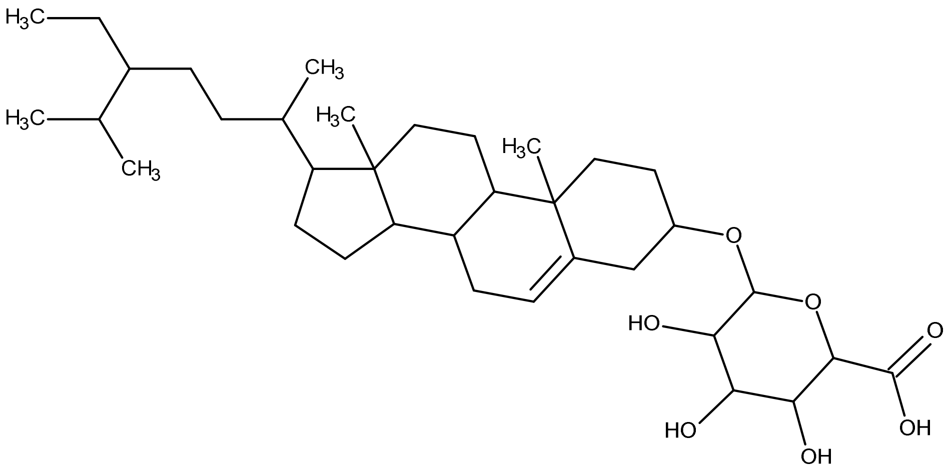

(26)

Figure S2. *(Continued)*

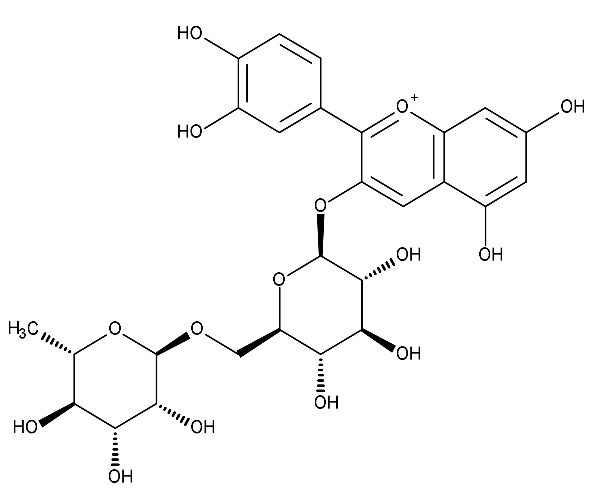

(27)

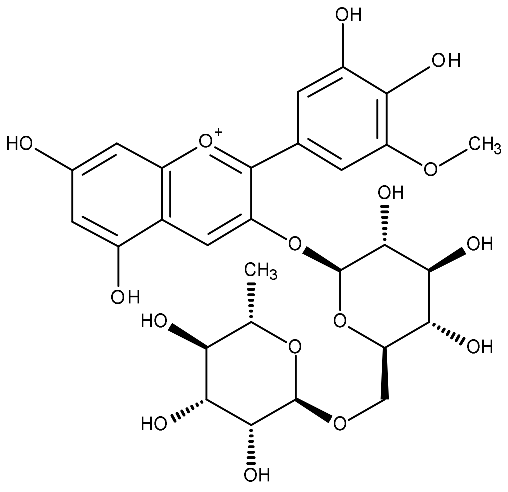

(28)

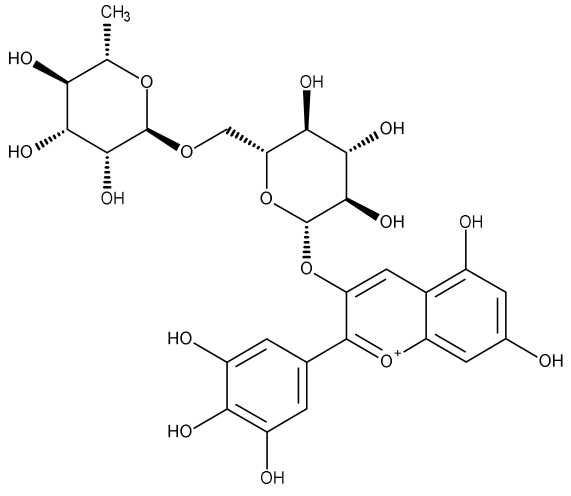

(29)

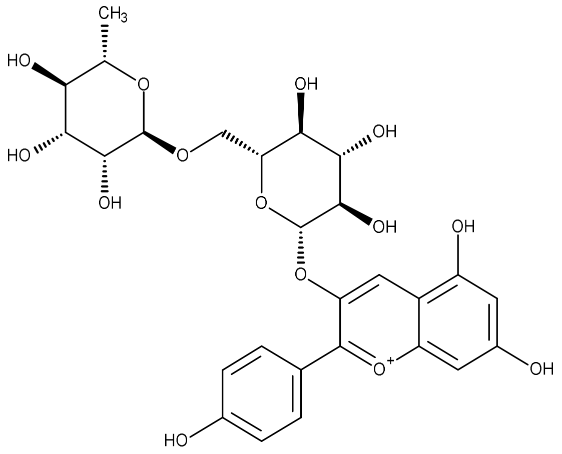

(30)

Figure S2. *(Continued)*

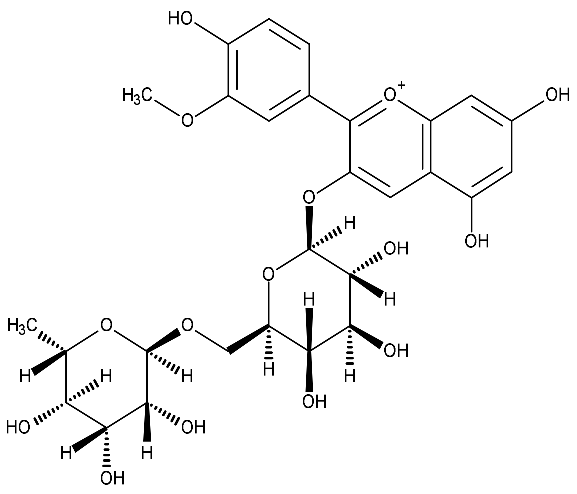

(31)

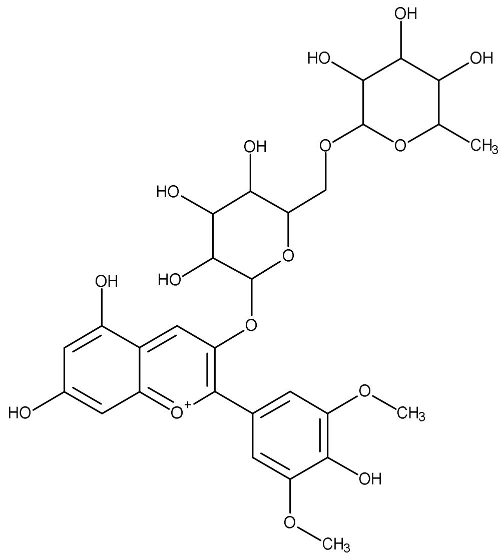

(32)

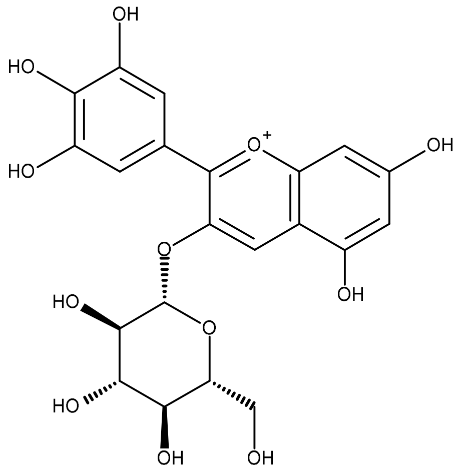

(33)

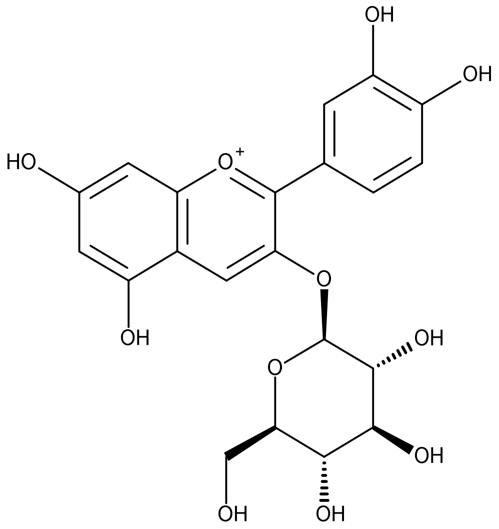

(34)

Figure S2. *(Continued)*

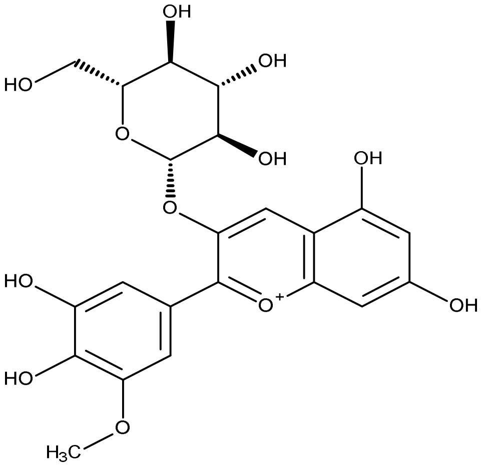

(35)

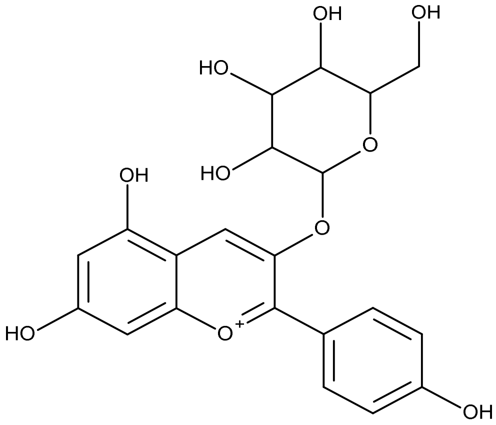

(36)

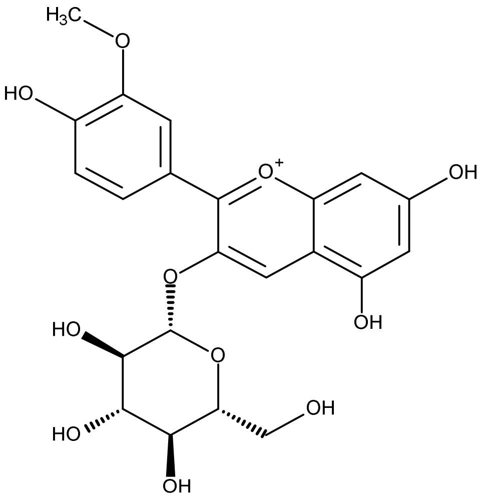

(37)

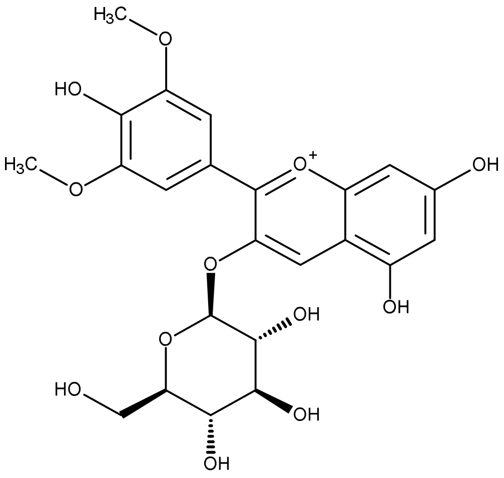

(38)

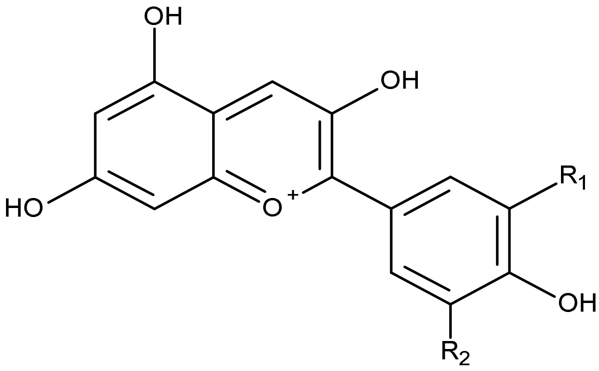

	R_1	R_2
(39):	OH	OH
(40):	OH	H
(41):	OH	OCH_3
(42):	H	H
(43):	OCH_3	H
(44):	OCH_3	OCH_3

Figure S2. *(Continued)*

Table S1: name of compounds of figure S2.

Name of compound	Compound number
Quercetin-3-O-rhamninoside	1
Kaempferol-3-O-rhamninoside	2
Quercetin-4′-O-rhamninoside	3
Kaempferol-4′-O-rhamninoside	4
Rhamnetin-3-O-rhamninoside	5
Rhamnocitrin-3-O-rhamninoside	6
Rhamnocitrin-4′-O-rhamninoside	7
Kaempferol	8
Quercetin	9
Isorhamnetin	10
Rhamnetin	11
Rhamnazin	12
Kaempferol-3-O-isorhamninoside	13
Rhamnocitrin-3-O-isorhamninoside	14
Rhamnetin-3-O-isorhamninoside	15
Emodin	16
Rhein	17
Chrysophanol	18
Physcion	19
1,4,6,8 tetrahydroxy-3 methyl anthraquinone	20
1-O-β-D-glucopyranosyl-4,6-di-O-α-L-rhamnopyran oside	
1,2,6,8 tetrahydroxy-3 methyl anthraquinone 8-O-β-D-glucopyranoside	21
1, 6 dihydroxy-3 methyl 6 [2′-Me (heptoxy)] anthraquinone	22
Physcion-3-O-β-rutinoside	23
Emodin-6O-α-L-rhamnopyranoside	24
β-sitosterol	25
β-sitosterol-3-O-β-D-glycopyranoside	26
Cyanidin 3-rutinoside	27
Petunidin 3-rutinoside	28
Delphinidin 3-rutinoside	29
Pelargonidin 3-rutinoside	30
Peonidin 3-rutinoside	31
Malvidin 3-rutinoside	32
Delphinidin 3-glucoside	33
Cyanidin 3-glucoside	34
Petunidin 3-glucoside	35
Pelargonidin 3-glucoside	36
Peonidin 3-glucoside	37
Malvidin 3-glucoside	38
Delphindin	39
Cyandin	40
Petunidin	41
Pelagonidin	42
Peonidin	43
Malvidin	44
